# Identification of the Constituents of Percutaneous Absorption from *Duhaldea nervosa* Based on UHPLC-Q-Exactive Orbitrap MS and Microdialysis Technique

**DOI:** 10.1155/2019/8328942

**Published:** 2019-11-11

**Authors:** Lianghong Liu, Lian Zhu, Kaiyan Gong, Xinjun Zhi, Ying Guan, Wei Cai

**Affiliations:** ^1^School of Pharmaceutical Sciences, Hunan University of Medicine, Huaihua, China; ^2^Hunan Province Key Laboratory for Antibody-Based Drug and Intelligent Delivery System, Hunan University of Medicine, Huaihua, China; ^3^Hunan Provincial Key Laboratory of Dong Medicine, Hunan University of Medicine, Huaihua, China

## Abstract

*Duhaldea nervosa* (*D. nervosa*) has been used for treatment of bone fracture by external use. Thus, the percutaneous absorption was crucial to the effect of *D. nervosa,* especially the constituents of percutaneous absorption. However, the constituents *in vivo* were never investigated to date. In this study, an efficient method was developed for the identification of constituents of percutaneous absorption using UHPLC-Q-Exactive Orbitrap MS and microdialysis technique. A total of 20 constituents including 15 chlorogenic acid analogues, 3 amino acids, and 2 organic acids were unambiguously or tentatively identified based on high-resolution mass data including MS and MS^2^, chromatography retention time, and bibliography data. To the best of our knowledge, this is the first study to report the constituents of percutaneous absorption from *D. nervosa*, which will be very helpful for understanding the bioactive compounds and quality control.

## 1. Introduction

*Duhaldea nervosa* (Wallich ex Candolle) A. Anderberg (*D. nervosa*), which belongs to the plant family Compositae, known as Maoxiucai or Xiaoheiyao in China, is a perennial plant widely distributed in the southwestern region of China and Southeast Asia [1]. Traditionally, it has been used as folk medicine in dispelling wind-chill, alleviating pain, promoting the circulation in meridian and collateral for treating migraine, rheumatism, and traumatic injury, especially in accelerating the healing of a fracture by external use or oral administration [2, 3]. Previous studies showed that the main constituents of this plant are steroids, terpenes, flavones, and chlorogenic acid analogues, which possess a variety of biological activities including anti-inflammatory activity [4–6].

Considering the usage of *D. nervosa*, which has been used as an external drug for the treatment of bone fracture, the percutaneous absorption was crucial to the effect of *D. nervosa,* especially the constituents of percutaneous absorption [7–9]. As far as we know, the constituent *in vivo* has not been investigated; therefore, it is worthwhile to identify the constituents of percutaneous absorption after *D. nervosa* for external use.

In recent decades, microdialysis has become a very powerful sampling technique that enables monitoring of the small molecules in *vivo* due to its excellent versatility [10, 11]. However, it is limited by the method of analyzing the resulting dialysate. LC-MS as a new technique was used to analyze and identify the constituent in extract botanical and biological sample including dialysate. Among all exiting platforms, ultra-high performance liquid chromatography (UHPLC) coupled with high resolution mass spectrometry (HRMS), including UHPLC-Q-TOF MS, UHPLC LTQ-Orbitrap MS, and UHPLC Q-Exactive Orbitrap MS, is the most powerful technique for the detection and identification of constituents, as UHPLC can provide a fast and effective separation while HRMS can provide accurate mass measurement and fragment ion, which will be very much beneficial for structure elucidation [12–14].

The present study was designed to detect and identify the main constituents of percutaneous absorption from *D. nervosa* by UHPLC-Q-Exactive Orbitrap MS and microdialysis. Finally, a total of 20 constituents including 15 chlorogenic acid analogues, 3 amino acids, and 2 organic acids were detected and identified based on high-resolution mass data including MS and MS^2^, chromatography retention time, and bibliography data. To the best of our knowledge, this is the first study to report the constituents of percutaneous absorption from *D. nervosa*, which will be very helpful for understanding the bioactive compounds and quality control.

## 2. Material and Methods

### 2.1. Chemicals and Materials

Acetonitrile and formic acid of LC-MS grade and methanol of LC grade were obtained from Aladdin Industrial Corporation. The ultrapure water used throughout the experiment was purified by a Milli-Q water purification system (Millipore, Milford, MA, United States). Other reagents of analytical grade were obtained from Aladdin Industrial Corporation.

The reference standards of 3-caffeoylquinic acid (3-CQA, neochlorogenic acid, X-014-170309), 4-caffeoylquinic acid (4-CQA, cryptochlorogenic acid, Y-067-180425), 5-caffeoylquinic acid (5-CQA, CGA, L-007-171216), 3,5-dicaffeoylquinic acid (3,5-DiCQA, isochlorogenic acid A, Y-068-170903), 3,4-dicaffeoylquinic acid (3,4-DiCQA, isochlorogenic acid B, Y-069-180105), and 4,5-dicaffeoylquinic acid (4,5-DiCQA, isochlorogenic acid C, Y-070-170515) were purchased from ChengDu Herbpurify Co., Ltd. (ChengDu, China). The purities of all reference standards were no less than 98% based on HPLC-UV analysis.

### 2.2. Sample Preparation

The root of *D. nervosa* was extracted by reflux with about eightfold 50% ethanol at 70°C for two hours. Then, the filtrate extracts were concentrated under reduced pressure to yield a black residue. Finally, the residue was redissolved in 50% ethanol to give a sample with a concentration of 0.5 g/mL.

### 2.3. Animal Experiments

All *in vivo* microdialysis were performed with CMA 402 syringe pump and MAB 85 refrigerated fraction collector (CMA, Microdialysis AB, Sweden). Four male SD rats (weighing 150–200 g, Hunan SJA Laboratory Animal Company, China) were used in *in vivo* study. All procedures were performed under the conditions of National Act on the Use of Experimental Animals. Anesthesia was induced by an intraperitoneal injection of 1.2 mL/100 g 20% urethane before each experiment. The leg of the rat was shaved carefully with razor without breaking the cuticle. After shaving, the rats were placed on an animal heat insulator. Body temperature was kept at 36–38°C. A CMA 20 Elite microdialysis probe (4 mm, polyarylethersulfone membrane, MWCO of 20 kDa) was then inserted into the dermis after fixing the introducer needle, parallel to the skin on the leg. Probe was perfused with normal saline before the insertion. The inlet and outlet were sealed to keep air from entering the probes.

The probes were perfused with normal saline at a flow rate of 2.0 *μ*L/min. A 60 min blank dialysate sample was collected for release of the insertion microtrauma prior to the application of *D. nervosa*. Then, a dosage of 5 g/mL of the material was applied to an area of 2.0 × 3.0 cm^2^ and covered with gauze and bandages. Microdialysate samples were obtained every 60 min up to 10 h, which was combined into one sample for each rat. During sampling, the microdialysis vials were cooled to 4°C; afterward, they were stored at −80°C until analysis.

### 2.4. Sample Pretreatment

All the microdialysates were pretreated by a solid-phase extraction (SPE) method. A SPE column (WondaSep C18, 200 mg/3 mL) was activated and equilibrated with 6 mL of methanol and 6 mL of water containing 0.5% formic acid, successively. A total of 1 mL microdialysate was loaded on the column. Then, the column was washed by 3 mL of water containing 0.5% formic acid and 3 mL of methanol, respectively. The methanol elute was collected and concentrated under N_2_ at room temperature to gain residue, which was redissolved in 100 *μ*L of acetronitrile-water (1 : 1, v/v) and centrifuged at 12000 rpm for 30 min at 4°C. Finally, an aliquot of 5 *μ*L supernatant was injected into the UHPLC-Q-Excative Orbitrap MS.

### 2.5. Instruments and Conditions

An Ultimate 3000 focused (Dionex, Sunnyvale, CA, USA) system equipped with an online vacuum degasser, a quaternary pump, and an auto sampler was used for UHPLC analysis. Separation was performed on an HYPERSIL GOLD C18 column (100 × 2.1 mm, 1.9 *μ*m) at 35°C. The mobile phase consisted of water containing 0.1% formic acid (A) and acetonitrile (B) using a gradient elution at a flow rate of 0.3 mL/min. The flowing gradient was applied: 5%–10% B at 0–2 min; 10%–20% B at 2–5 min; 20%–25% B at 5–10 min; 25%–55% B at 10–12 min; 55%–80% B at 12–15 min; 80%–5% B at 15–16 min; and 5% B at 16–20 min.

All ESI-MS^*n*^ analyses were carried out on a Q-Exactive Focus Orbitrap MS (Thermo Electron, Bremen, Germany) coupled with a heated electrospray ionization source (Thermo Electron, Bremen, Germany) in the negative mode. The tune operating parameters were as follows: the rate of sheath gas flow and auxiliary gas flow was 30 and 10 (arbitrary unit), respectively; spray voltage, 3.0 kV; the temperature of capillary and auxiliary gas heater was 320°C and 350°C, respectively; high-resolution MS^*n*^ was operated at full scan with a mass range of m/z 100–1200 at a resolution of 35000 and MS^2^ at a resolution of 17500 triggered by data-dependent MS^*n*^ scanning; nitrogen served as collision gas; and the energy was set as normalized collision energy 30%.

### 2.6. Data Processing and Analysis

The Xcalibur software version 4, (Thermo Fisher Scientific, San Jose, CA, USA) was used to acquire the raw data including the full-scan MS and MS^2^ data, which were processed by the Compound Discover version 3 using the metabolomics workflow templates to detect the differential components between the microdialysates before and after application of *D. nervosa*. The detailed parameters of metabolomics workflow template were as follows: The minimum peak intensity was set as 10000; the maximum element counts were C30 H60 O20 S4 N10 Cl4; the mass tolerance of MS and MS^2^ was within 5 and 10 ppm, respectively; and differential analysis was selected for postprocessing.

## 3. Results and Discussion

### 3.1. Identification of Percutaneous Absorption Constituents of *D. nervosa*

**Figure 1:**
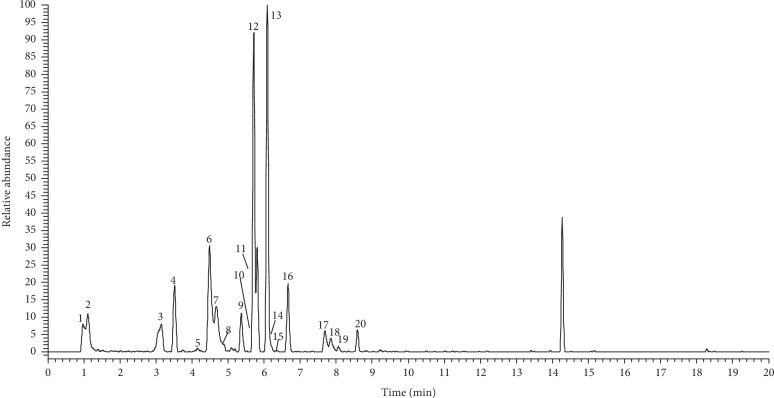
The high-resolution extracted ion chromatography of multiple constituents at m/z 133.01425, 172.09792, 203.08260, 337.09289, 353.08781, 367.10345, and 515.11950.

A total of 20 constituents were detected and identified based on UHPLC-Q-Exactive Orbitrap MS and microdialysis technique. The retention time and mass spectrometric data of those constituents are listed in Table 1. The high-resolution extracted ion chromatography of those compounds is shown in Figure 1.

### 3.2. Identification of Chlorogenic Acid Analogues

Compounds 3, 6, 7, 17, 18, and 20 with the pseudomolecular ion [M-H]^−^ of m/z 353.08783 (0.07 ppm, C_16_H_17_O_9_), 353.08771 (−0.27 ppm, C_16_H_17_O_9_), 353.08780 (−0.02 ppm, C_16_H_17_O_9_), 515.11981 (0.60 ppm, C_25_H_23_O_12_), 515.11938 (−0.23 ppm, C_25_H_23_O_12_), and 515.11902 (−0.93 ppm, C_25_H_23_O_12_), respectively, were accurately identified as tran-3-CQA, tran-5-CQA, tran-4-CQA, 3,4-DiCQA, 3,5-DiCQA, and 4,5-DiCQA by comparing their chromatography retention times, accurate mass measurement, and fragment pattern with those data of reference compounds.

Compounds 9, 11, and 19 were eluted at 5.37, 5.69, and 8.16 min, respectively, with the deprotonation ion [M-H]^−^ of m/z 353.08783 (0.07 ppm, C_16_H_17_O_9_), 515.11969 (0.37 ppm, C_25_H_23_O_12_), and 515.11920 (−0.58 ppm, C_25_H_23_O_12_), suggesting that those are isomer of compounds 3, 6, 7 and 17, 18, 20, respectively, which were confirmed by the MS^2^ spectrum. Finally, those were tentatively characterized as cis-5-CQA, 1,3-DiCQA, and 1,5-DiCQA according to the literature data [15, 16]. Compounds 5, 10, and 15 possessing a [M-H]^−^ ion of m/z 337.09323 (1.01 ppm, C_16_H_17_O_8_), 337.09302 (0.38 ppm, C_16_H_17_O_8_), and 337.09286 (−0.09 ppm, C_16_H_17_O_8_), respectively, were eluted at 4.12, 5.59, and 6.31 min, respectively, suggesting that they were *p*-coumaroylquinic acid (*p*CoQA). Considering the base peak at m/z 163.0389, 191.0552, and 173.0445 of the precursor ion at m/z 337 [17, 18], they were tentatively presumed to be 3-*p*CoQA, 5-*p*CoQA, and 4-*p*CoQA, respectively.

Compounds 8, 14, and 16 eluted at 4.70, 6.13, and 6.64 min, respectively. All of them showed a pseudomolecular ion [M-H]^−^ of m/z 367.10306 (−1.08 ppm, C_17_H_19_O_9_), 367.10355 (0.26 ppm, C_17_H_19_O_9_), and 367.10294 (−1.40 ppm, C_17_H_19_O_9_), respectively. The base peak at 193.0497, 173.0445, and 191.0547 in MS^2^ spectrum were used to discriminate isomers of feruloylquinic acid (FQA) [17, 18]; therefore, they were tentatively inferred to be 3-FQA, 4-FQA, and 5-FQA, respectively.

### 3.3. Identification of Amino Acid

Compounds 4, 12, and 13 were detected at 3.45, 5.70, and 6.07 min with the [M-H]^−^ ion at m/z 203.08181 (−3.55 ppm, C_11_H_11_O_2_N_2_), 172.09697 (−5.05 ppm, C_8_H_14_O_3_N), and 172.09691 (−5.85 ppm, C_8_H_14_O_3_N), respectively. The MS^2^ spectrum was used to elucidate the structure by searching various MS/MS databases including massbank, METLIN, and KEGG [19]. Therefore, they were temporarily determined to be tryptophan, N-acetyl-leucine, and N-acetyl-alloisoleucine, respectively.

### 3.4. Identification of Organic Acid

Compounds 1 and 2 with the same pseudomolecular ion [M-H]^−^ of m/z 133.014 (C_4_H_5_O_5_) were detected at 0.96 and 1.11 min, respectively. The fragment ion at 115.0026 (−9.4 ppm, C_4_H_3_O_4_), 71.0131 (−10.6 ppm, C_3_H_3_O_2_) and 89.0235 (−10.3 ppm, C_3_H_5_O_3_) was detected in MS^2^ spectrum by natural loss of H_2_O, CH_2_O_3_, and CO_2_, suggesting the presence of carboxyl and hydroxyl. According to the literature [20], they were temporarily identified as malic acid isomers.

## 4. Conclusion

The constituents of percutaneous absorption following the external use of *D. nervosa* were investigated using UHPLC-Q-Exactive Orbitrap MS and microdialysis technique. Finally, a total of 20 constituents including 15 chlorogenic acid analogues, 3 amino acids, and 2 organic acids were detected and identified based on high-resolution mass data including MS and MS^2^, chromatography retention time, and bibliography data. To our best knowledge, this is the first study to investigate the constituents of percutaneous absorption from *D. nervosa*, which will be very beneficial for understanding the bioactive compounds and quality control.

## Figures and Tables

**Table 1:** The retention time and mass spectrometric data of constituents of percutaneous absorption.

Peak	*t*_R_	Theoretical mass	Experimental mass	Error	Formula [M-H]^−^	Fragment	Identification
1	0.96	133.01425	133.01361	−4.78	C_4_H_5_O_5_	MS^2^ [133]: 115.0026 (100), 71.0131 (42), 89.0235 (8)	Malic acid isomer
2	1.11	133.01425	133.01365	−4.49	C_4_H_5_O_5_	MS^2^ [133]: 115.0025 (100), 71.0131 (43), 89.0236 (6)	Malic acid isomer
3	3.15	353.08781	353.08743	−1.06	C_16_H_17_O_9_	MS^2^ [353]: 191.0547 (100), 179.0336 (76), 135.0436 (21)	3-CQA
4	3.45	203.08260	203.08188	−3.55	C_11_H_11_O_2_N_2_	MS^2^ [203]: 116.0492 (100), 74.0233 (38), 142.0650 (27), 159.0918 (21)	Tryptophan
5	4.12	337.09289	337.09323	1.01	C_16_H_17_O_8_	MS^2^ [337]: 163.0389 (55), 119.0489 (20), 191.0552 (9)	3-*p*CoQA
6	4.50	353.08781	353.08771	−0.27	C_16_H_17_O_9_	MS^2^ [353]: 191.0547 (100)	5-CQA
7	4.67	353.08781	353.08780	−0.02	C_16_H_17_O_9_	MS^2^ [353]: 173.0441 (100), 179.0336 (82), 191.0547 (52), 135.0435 (30)	4-CQA
8	4.70	367.10345	367.10306	−1.08	C_17_H_19_O_9_	MS^2^ [367]: 193.0497 (100), 134.0361 (18)	3-FQA
9	5.37	353.08781	353.08783	0.07	C_16_H_17_O_9_	MS^2^ [353]: 191.0548 (100), 135.0435 (4), 179.0334 (3), 173.0078 (3),	Cis-5-CQA
10	5.59	337.09289	337.09302	0.38	C_16_H_17_O_8_	MS^2^ [337]: 191.0552 (100), 173.0445 (10), 163.0390 (8)	5-*p*CoQA
11	5.69	515.11950	515.11969	0.37	C_25_H_23_O_12_	MS^2^ [515]: 191.0545 (100), 179.0333 (88), 353.0860 (16), 135.0434 (13)	1,3-DiCQA
12	5.70	172.09792	172.09697	−5.50	C_8_H_15_NO_3_	MS^2^ [172]: 130.0861 (100)	*N*-Acetyl-leucine
13	6.07	172.09792	172.09691	−5.85	C_8_H_15_NO_3_	MS^2^ [172]: 130.0861 (100)	*N*-Acetyl-alloisoleucine
14	6.13	367.10345	367.10355	0.26	C_17_H_19_O_9_	MS^2^ [367]: 173.0445 (100), 93.0331 (88), 191.0552 (47)	4-FQA
15	6.31	337.09289	337.09286	−0.09	C_16_H_17_O_8_	MS^2^ [337]: 173.0445 (100)	4-*p*CoQA
16	6.64	367.10345	367.10294	−1.40	C_17_H_19_O_9_	MS^2^ [367]: 191.0547 (100)	5-FQA
17	7.69	515.11950	515.11981	0.60	C_25_H_23_O_12_	MS^2^ [515]: 173.0441 (100), 179.0335 (89), 191.0547 (34), 135.0435 (14), 353.0865 (14)	3,4-DiCQA
18	7.85	515.11950	515.11938	−0.23	C_25_H_23_O_12_	MS^2^ [515]: 191.0546 (100), 179.0335 (62), 353.0865 (15), 135.0435 (11)	3,5-DiCQA
19	8.16	515.11950	515.11920	−0.58	C_25_H_23_O_12_	MS^2^ [515]: 191.0546 (100), 179.0335 (72), 353.0865 (18), 173.0445 (11), 135.0435 (11)	1,5-DiCQA
20	8.62	515.11950	515.11902	−0.93	C_25_H_23_O_12_	MS^2^ [515]: 173.0445 (100), 179.0340 (71), 191.0552 (25), 353.0865 (22), 135.0439 (8)	4,5-DiCQA

## Data Availability

The data used to support the finding of this study are available from the corresponding author upon request.
